# Unilateral Cystic Kidney Disease in a Young Female

**DOI:** 10.7759/cureus.24719

**Published:** 2022-05-04

**Authors:** Nicholas W Salupo, Megan Grant, Shashikant Patel

**Affiliations:** 1 Internal Medicine, Grandview Medical Center, Dayton, USA; 2 Nephrology, Grandview Medical Center, Dayton, USA

**Keywords:** genetic renal diseases, general internal medicine, autosomal-dominant polycystic kidney disease, general nephrology, unilateral renal cystic disease

## Abstract

Unilateral renal cystic disease has been mostly reported in older male patients; however, this case is novel as the youngest reported case in the literature and in a female patient. We present a 22-year-old female with no past medical history and no family history of renal disease that was incidentally found to have unilateral renal cystic disease on computed tomography imaging. The patient’s renal function was not impaired and the cystic kidney was found to be functioning appropriately on an intravenous pyelogram. The unilateral cystic disease is benign but must be differentiated from autosomal dominant polycystic disease to prevent morbidity and mortality.

## Introduction

Unilateral renal cystic disease (URCD) of the kidney is a non-familial and non-progressive multicystic kidney disorder, characterized by the replacement of the renal parenchyma with a cluster of multiple cysts and an unaffected contralateral kidney. This rare condition is distinct from autosomal dominant polycystic kidney disease (ADPKD) and is generally a benign condition with preserved renal function [1,2]. The cases reported in the literature suggest this condition most frequently affects males after the fifth decade of life; however, we report a case of a young female who was found to have unilateral cysts with preserved renal function [2].

## Case presentation

**Figure 1:**
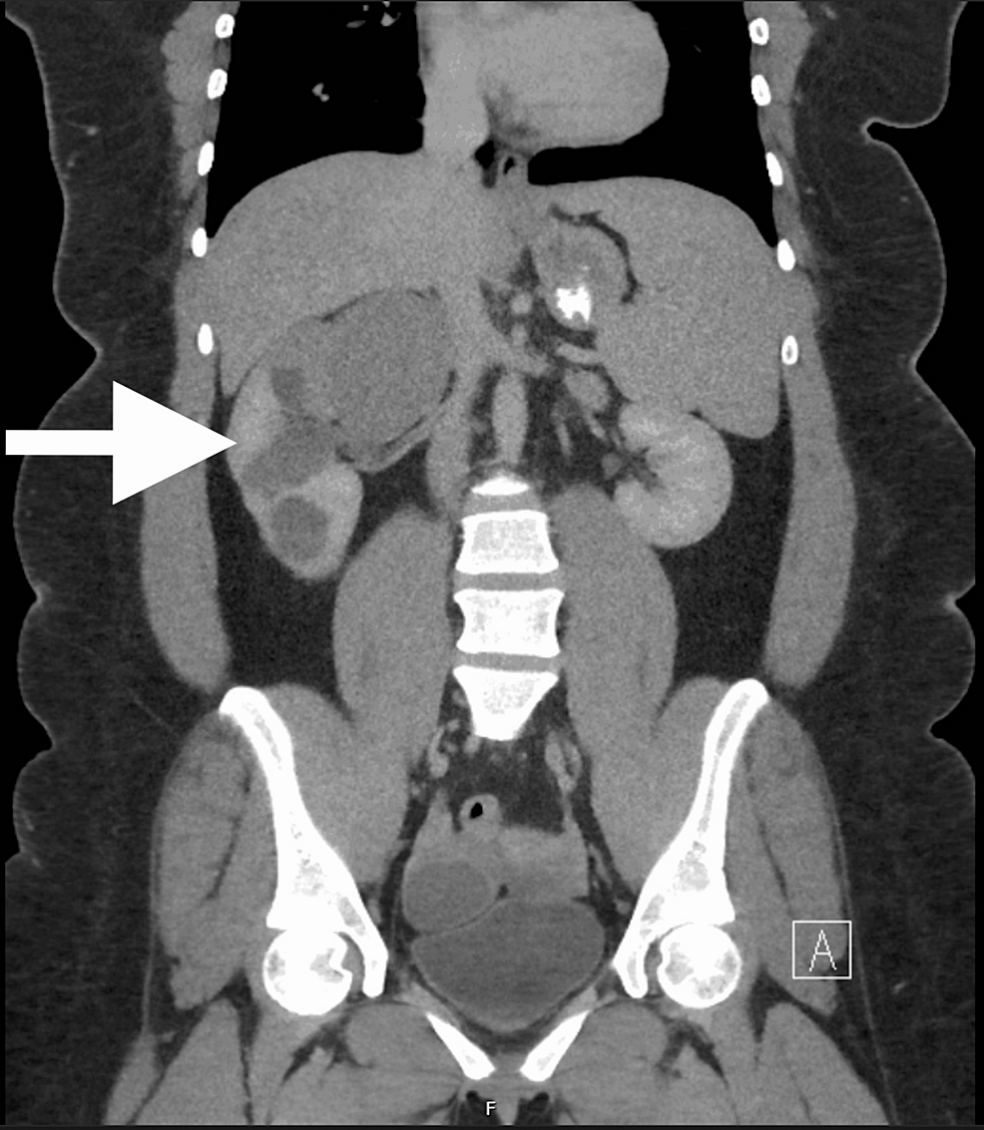
Abdominal computed tomography scan demonstrating multiple cortical and renal sinus cysts in the right kidney. No involvement of the left kidney was identified.

A 22-year-old obese female presented to the emergency department complaining of intermittent, cramping, non-radiating abdominal pain localized to the right upper quadrant. The pain was worse with deep inspiration. She endorsed associated nausea and occasional non-bilious, non-bloody emesis. She reported normal bowel movements. She was on her menstrual cycle but denied pelvic pain. There was no family history of renal disease or family members on dialysis. Her blood pressure was 157/84 and her heart rate was 109 beats per minute. Serum creatinine was 1.2 mg/dL. Right upper quadrant ultrasound showed no evidence of cholelithiasis or acute cholecystitis but a markedly enlarged kidney with innumerable simple and complex cystic lesions. A CT scan of the abdomen and pelvis showed multiple cortical and renal sinus cysts of varying attenuation in the right kidney and a normal left kidney (Figure 1).

The patient was discharged after supportive care and given a referral to nephrology for the incidental CT findings.

**Figure 2:**
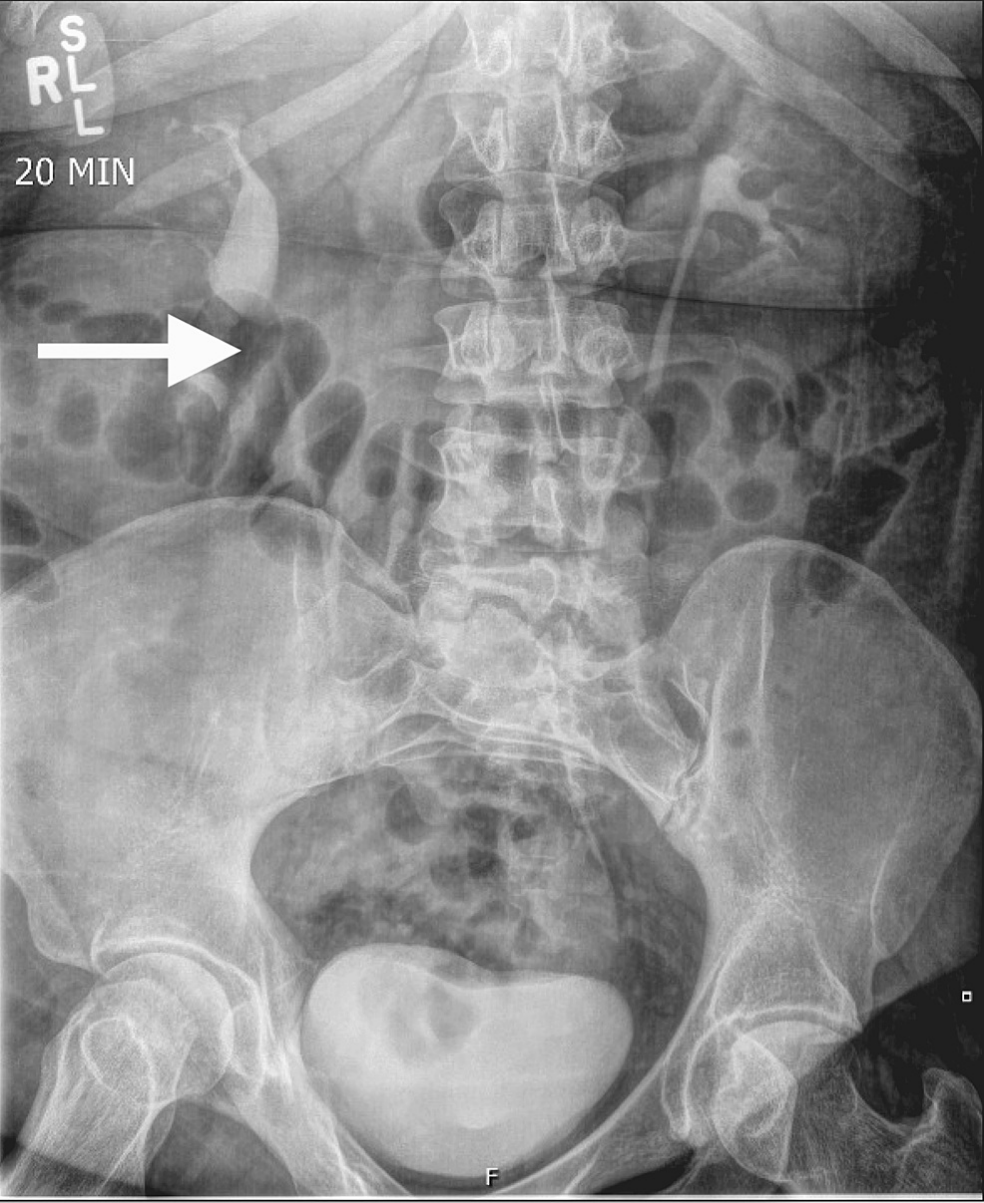
IV Pyelogram showing symmetric excretory function of the kidneys bilaterally. There is mass effect visualized in the right kidney without obstruction. The ureters are slender bilaterally.

She presented to the nephrology clinic approximately four months later for evaluation. She was hypertensive with a blood pressure of 139/75 otherwise the interval history and physical exam were unchanged. Serum creatinine was 0.9 mg/dL. An intravenous pyelogram showed prompt symmetric excretory function of the kidneys. Contour of the right kidney was slightly altered relative to the sites of the cystic masses indicating mass effect. The calyces in the lower pole of the right kidney were effaced but not particularly dilated. The left kidney was normal in size and contour. The ureters were slender bilaterally, bladder contour was maintained, and voiding was complete (Figure 2).

No further interventions were performed and she continues routine follow-up with her primary care physician without complication.

## Discussion

URCD was first described in 1964 by Bergman and Nehm [3]. However, it was not until 1989 that it was described as a unique entity separate from ADPKD [4]. The pathogenesis is currently unknown [5]. There are less than 100 cases reported in the literature, which suggests this is either a rare condition or, more likely, it is a benign condition that remains asymptomatic or goes undiagnosed [6]. Clinical presentation is commonly associated with hematuria, hypertension, abdominal mass, and flank pain [6]. This case is novel because it is reported both in a female and she is in her second decade of life. It is important to distinguish between URCD and ADPKD because ADPKD requires dramatic intervention to prevent serious morbidity and mortality.

The Bosniak classification of renal cysts exists to differentiate benign from malignant cystic masses of the kidney. The most recent update, the Bosniak classification, version 2019, recently expanded criteria from computed tomography imaging alone to include magnetic resonance imaging of the kidneys. The Bosniak Classification, version 2019 defines a cystic renal mass as a mass that, based on subjective visual inspection, is composed of less than approximately 25% enhancing components [7]. Masses with greater than 25% enhancing components are more likely to represent solid masses that typically behave more aggressively and therefore have more aggressive management. Class 1 masses are well-defined, thin (≤ 2 mm) smooth-walled, without septa or calcifications, and contain simple fluid (-9 to 20 HU). Class II masses consist of six subtypes but generally are well defined with thin (≤ 2 mm) smooth walls, may contain 1-3 septa, and may demonstrate homogenous enhancement. Class IIF masses demonstrate smooth with minimally thickened (3 mm) enhancing walls, one or more smooth minimally thickened (3 mm) septa, or many (>4) smooth thin (≤ 2 mm) enhancing septa. Class III masses show one or more enhancing thick (≥ 4 mm width) or enhancing irregular walls or septa. Class IV masses show one or more enhancing nodules [8]. Bosniak I and II masses can be considered benign without further evaluation, IIF is probably benign but warrant a follow-up, and class III and IV require surgical evaluation.

The differential diagnosis for renal cysts includes ADPKD, autosomal recessive polycystic kidney disease (ARPKD), von Hippel-Landau disease, tuberous sclerosis, reflux nephropathy, and end-stage renal disease. ARPKD is less likely in this case as it is a disease affecting one in 20,000 neonatal and pediatric patients and presents in the first year of life [9]. Von Hippel-Lindau disease most commonly presents with hemangioblastomas of the spine, brain, and eye but is also associated with simple or complex renal cysts, solid renal tumors (renal cell carcinoma), and pheochromocytoma. Tuberous sclerosis is less likely in this case given the absence of skin manifestations that affect almost every patient with tuberous sclerosis. Reflux nephropathy is another hereditary disease of the kidney most commonly affecting young women. It typically presents hypertension, proteinuria, and urinary tract infections [10]. There is the rare possibility that this case represents unilateral ADPKD; however, the patient does not currently show signs of extrarenal manifestations such as hepatic or pancreatic cysts, diverticulosis coli, or saccular aneurysm [11]. The patient described in this case, due to financial barriers, declines to proceed with genetic testing for PKD1, PKD2, and PKHD1 therefore we are unable to confirm benign URCS. Again, this patient has no family history of renal disease making an inherited disease unlikely. She has been compliant with continued follow up, her creatinine has remained within normal limits, and there has been no new proteinuria. In cases of non-ADPKD, surveillance is still requires because of the non-zero risk of unilateral cysts evolving into complicated cysts, progressing to bilateral disease, or developing in malignancy [12].

## Conclusions

Primary care physicians and hospitalists should be aware of this benign renal cystic condition. It still warrants referral to a nephrologist to rule out nephritic, nephrotic, and malignant conditions. However, when correctly diagnosed URCD does not require the costly and invasive intervention that ADPKD demands. There is no consensus on what constitutes appropriate follow-up but yearly biochemical measurements of renal function, urinalysis, and renal ultrasound are likely sufficient to monitor for advancing disease however URCD is not likely to progress to non-benign conditions.

## References

[1] Choh NA, Rashid M (2010). Unilateral renal cystic disease. Indian J Nephrol.

[2] Hwang DY, Ahn C, Lee JG (1999). Unilateral renal cystic disease in adults. Nephrol Dial Transplant.

[3] Bergman H, Nehme DA (1964). Unilateral polycystic renal disease. N Y State J Med.

[4] Cho KJ, Thornbury JR, Bernstein J, Heidelberger KP, Walter JF (1979). Localized cystic disease of the kidney: angiographic-pathologic correlation. Am J Roentgenol.

[5] Slywotzky CM, Bosniak MA (2001). Localized cystic disease of the kidney. AJR Am J Roentgenol.

[6] Neyaz Z, Kumar S, Lal H, Kapoor R (2012). Localized cystic disease of the kidney: a rare entity. J Radiol Case Rep.

[7] Schieda N, Davenport MS, Krishna S (2021). Bosniak Classification of Cystic Renal Masses, Version 2019: A Pictorial Guide to Clinical Use. RadioGraphics.

[8] Silverman SG, Pedrosa I, Ellis JH (2019). Bosniak classification of cystic renal masses, version 2019: an update proposal and needs assessment. Radiology.

[9] Guay-Woodford LM, Desmond RA (2003). Autosomal recessive polycystic kidney disease: the clinical experience in North America. Pediatrics.

[10] Aeddula NR, Baradhi KM (2022). Reflux Nephropathy. https://www.ncbi.nlm.nih.gov/books/NBK526055/.

[11] Tandon A, Qureshi MS, Ahmad I, Singh UR, Bhatt S (2018). Unilateral autosomal dominant polycystic kidney disease with co-existent renal cell carcinoma: a rare entity. Egypt J Radiol Nucl Med.

[12] Miyamoto Y, Nagai M, Hirayama K, Shimohata H, Kobayashi M (2011). Non-hereditary multiple renal cysts in unilateral kidney. NDT Plus.

